# Development of a versatile and conventional technique for gene disruption in filamentous fungi based on CRISPR**-**Cas9 technology

**DOI:** 10.1038/s41598-017-10052-3

**Published:** 2017-08-23

**Authors:** Yan-Mei Zheng, Fu-Long Lin, Hao Gao, Gen Zou, Jiang-Wei Zhang, Gao-Qian Wang, Guo-Dong Chen, Zhi-Hua Zhou, Xin-Sheng Yao, Dan Hu

**Affiliations:** 1 Institute of Traditional Chinese Medicine and Natural Products, College of Pharmacy, Guangdong Province Key Laboratory of Pharmacodynamic Constituents of TCM and New Drugs Research, Jinan University, Guangzhou, 510632 China; 2 CAS-Key Laboratory of Synthetic Biology, Institute of Plant Physiology and Ecology, Shanghai Institutes for Biological Sciences, Chinese Academy of Sciences, Shanghai, 200032 China; 3 State Key Laboratory of Bioorganic and Natural Products Chemistry, Shanghai Institute of Organic Chemistry, University of Chinese Academy of Sciences, Shanghai, 200032 China

## Abstract

Filamentous fungi represent an invaluable source of pharmaceutically active compounds. The development of versatile methods to genetically manipulate filamentous fungi is of great value for improving the low yields of bioactive metabolites and expanding chemical diversity. The CRISPR-Cas9-based system has become a common platform for genome editing in a variety of organisms. However, recent application of this technology in filamentous fungi is limited to model strains, a versatile method for efficient gene disruption in different fungi is lacking. Here, we investigated the utility of the CRISPR-Cas9 system in a less-studied fungus *Nodulisporium* sp. (No. 65-12-7-1), and we have developed an efficient CRISPR-Cas9-based gene disruption strategy by simultaneous transformation of *in vitro* transcriptional gRNA and the linear maker gene cassette into the Cas9-expressing fungi. We found that the linear marker gene cassette could not only allow for selection of transformants, but also significantly enhance the gene disruption efficiency by inserting itself into the Cas9 cut site. Moreover, the above approach also demonstrated its efficiency in two other phylogenetically distinct strains *Aspergillus oryzae* NSAR1 and *Sporormiella minima* (No. 40-1-4-1) from two different classes of Ascomycota. These results suggested that a versatile CRISPR-Cas9-based gene disruption method in filamentous fungi was established.

## Introduction

Filamentous fungi serve as an invaluable source of biologically active natural products including the most widely used antibacterials penicillin and cephalosporin, the anti-fungals griseofulvin and echinocandin, and statins, which are a class of cholesterol-lowering agents^1^. The recent advancement of DNA sequencing technology has elucidated a number of fungal genome sequences, revealing a myriad of previously unknown gene clusters of potentially bioactive compounds. However, characterizations of these cryptic gene clusters are greatly hampered due to the lack of versatile methods for the genetic manipulation of filamentous fungi^2, 3^.

Genome-editing is a type of genetic engineering in which DNA is inserted, replaced, or removed from a genome in a sequence-specific manner. Recently, several approaches to genome editing have been developed, including zinc-finger nucleases (ZFNs)^4^, transcription activator-like effector nucleases (TALENs)^4, 5^ and the RNA-guided CRISPR-Cas9 nuclease system^6^. The first two technologies use a strategy of tethering endonuclease catalytic domains to modularly designed DNA-binding proteins for inducing DNA double-stranded breaks (DSBs) at specific sites. In contrast, the CRISPR-Cas9 technique is a RNA-guided system, in which the Cas9 nuclease is guided by a single guide RNA (gRNA), which introduces a DSB at the desired site. The break is then repaired by the non-homologous end joining (NHEJ) pathway, often resulting in short deletions and substitutions that may lead to frame shifts and generation of premature stop codons. Alternatively, a DNA repair template (donor DNA) can be simultaneously provided with the gRNA to activate the homology directed pathway (HDR), leading to the desired alterations in the genome^7^. Owing to its versatility and high efficiency, the CRISPR-Cas9 system is considered to be the most remarkable breakthrough in genome editing technology. To date, this technology has been successfully applied in a number of organisms including yeast, fishes, plants and mammalian cells^8, 9^.

Recently, the applications of CRISPR-Cas9 technology in filamentous fungi have drawn attention and their validity has been demonstrated in several fungal strains including *Pyricularia oryzae* ^10^, *A*. *fumigatus* ^11^, *A*. *oryzae* ^12^, *A*. *niger* ^13^, *Trichoderma reesei* ^14^, *Talaromyces atroroseus* ^15^, *Penicillium chrysogenum* ^16^, *Neurospora crassa* ^17^, *Ustilago maydis* ^18^, *Candida albicans* ^19^, *Alternaria alternata* ^20^. However, most of these studies are limited to model strains belonging to a relatively small subset of Ascomycota (such as *Aspergillus*, *Penicillium* and *Neurospora*) that have been thoroughly studied and are genetically tractable. Moreover, the validity and utility of the CRISPR-Cas9-based methods established in these studies are only evaluated in one strain or several strains from the same genus, thereby a versatile method for efficient gene disruption in a broad range of filamentous fungi is still lacking.

*Nodulisporium* is a seldom studied fungus genus, but known to produce a diverse of active natural products. In our previous study, biologically important demethoxyviridin and its derivatives were isolated from an endolichenic fungus *Nodulisporium* sp. (No. 65-12-7-1)^21, 22^. Most of these compounds exhibited substantial anti-Alzheimer’s disease activity. Thus, establishment of a genetic manipulation system in this fungus would be of great value to reveal the biosynthetic assembly of demethoxyviridin and its derivatives.

Here, we have developed an efficient and convenient CRISPR-Cas9-based gene disruption method in *Nodulisporium* sp. (No. 65-12-7-1) by simultaneous delivery of *in vitro* transcriptional gRNA and the linear selectable maker gene cassette into Cas9-expressing fungi. We found that the DNA fragment containing the maker gene cassette could not only function for selection of transformants, but also enhance significantly the gene disruption efficiency by inserting itself into the Cas9 cut site. Moreover, its utility has been demonstrated in three phylogenetically distinct strains, suggesting that a versatile gene disruption method in filamentous fungi has been established.

## Results

### Establishment of protoplast preparation and transformation for *Nodulisporium* sp. (No. 65-12-7-1)

**Figure 1:**
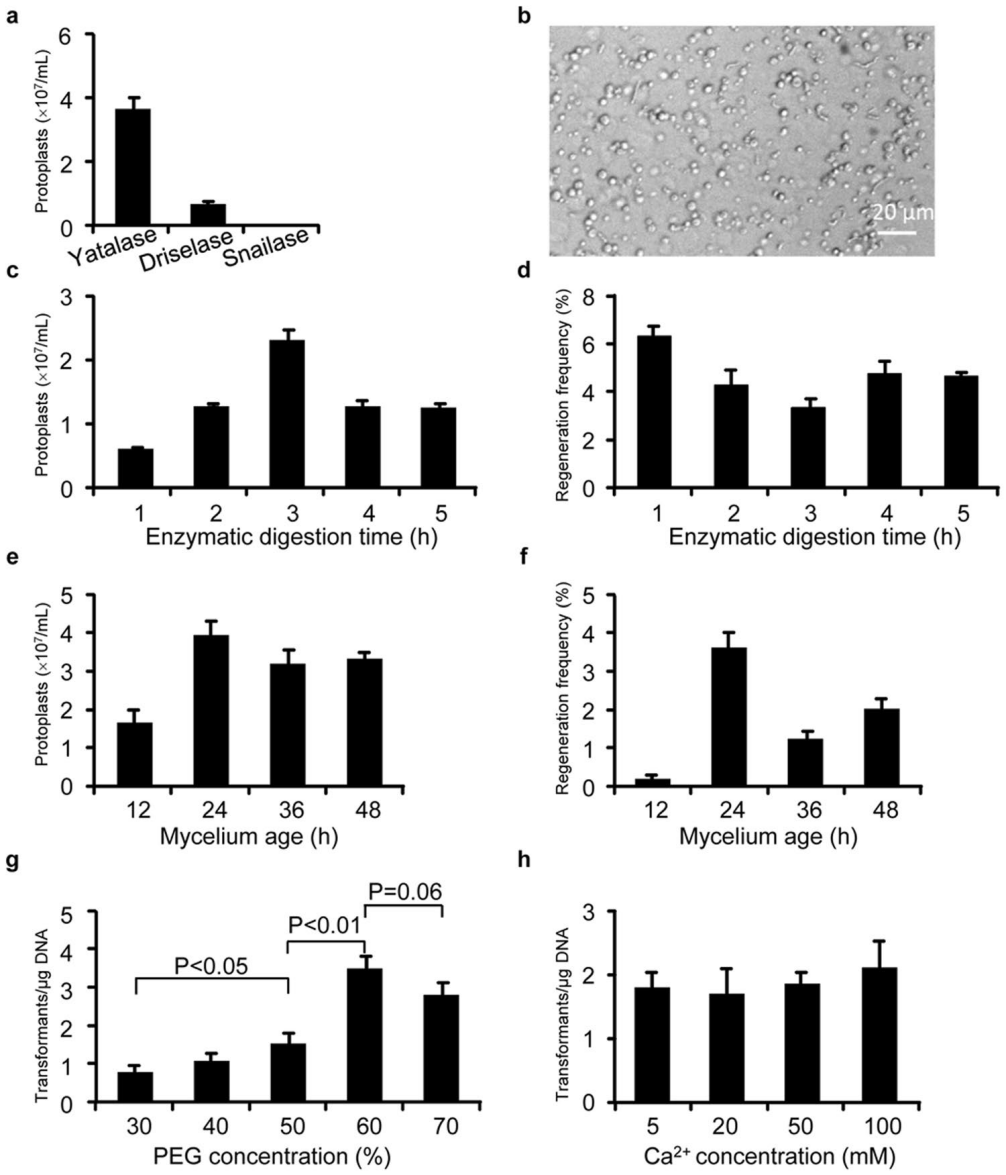
Protoplast preparation and transformation for *Nodulisporium* sp. (No. 65-12-7-1). (**a**) The amount of protoplasts produced by treatment of the mycelia of *Nodulisporium* sp. (No. 65-12-7-1) with 3 different enzymes: Yatalase (10 mg/mL), Driselase (10 mg/mL) and Snailase (10 mg/mL). (**b**) Microscopic check of protoplasts released from the mycelium of *Nodulisporium* sp. (No. 65-12-7-1). (**c**) The amount of protoplasts produced by Yatalase treatment for different incubation time. (**d**) The regeneration frequency of protoplasts produced by Yatalase treatment for different incubation time. (**e**) The amount of protoplasts produced from the mycelia of different age. (**f**) The regeneration frequency of protoplast produced from the mycelia of different age. (**g**) The transformation efficiency under different concentrations of PEG. Student’s t-test was performed for comparing the transformation efficiency under different concentrations of PEG. (**h**) The transformation efficiency under different concentrations of Ca^2+^. All values are means ± standard error of the mean from three independent experiments.

Since strains belonging to the *Nodulisporium* genus are seldom studied and most of them are not genetically tractable, establishment of the genetic transformation system for *Nodulisporium* sp. (No. 65-12-7-1) was our primary task. A stable and viable protoplast formation system is the basis for protoplast-based transformation. To prepare transformable protoplasts, several key factors including enzyme system, digestion time, and mycelium age were evaluated. Enzyme selection for cell wall digestion is the most important factor for protoplast formation because there is a great variation in cell wall structure among different fungi^23^. Here, we tested 3 different lytic enzymes in the protoplast preparation: Yatalase (TaKaRa, China), Driselase (Sigma-Aldrich, USA) and Snailase (Sangon Biotech Co., Ltd., China). Briefly, 1 g of mycelia (wet weight) were collected and suspended in 5 mL of enzyme solution (10 mg/mL) followed by incubation at 30 °C for 3 h. The number of the released protoplast was then counted under the microscope. We found that Yatalase was the most efficient enzyme in the release of the protoplast from the mycelia of *Nodulisporium* sp. (No. 65-12-7-1) (Fig. 1a). The release of the protoplast was verified using phase contrast microscopy (Fig. 1b). We then determined the optimal incubation time for Yatalase treatment by examining the release of protoplasts at different incubation times. As shown in Fig. 1c, the released protoplasts increased and reached a peak at 3 h, and then declined. Since exposure to the enzyme for too long could be harmful to the protoplasts^24^, the regeneration frequency was also investigated. The released protoplasts under different conditions were serially diluted by 100, 1000 and 10000 folds, and then planted on the M medium with 2% agar and 1.2 M sorbitol (osmotic stabilizer). As a negative control, protoplasts were also plated on the medium without osmotic stabilizer. The cultures were incubated at 28 °C for 5 d before colonies were counted. The results showed a maximal regeneration frequency at 1 h (Fig. 1d). Considering that the amount of protoplasts released at 1 h were already sufficient for subsequent transformation, we determined the optimal enzymolysis time to be 1 h.

The age of the mycelium used for enzyme treatment is another influential factor for protoplast isolation^25^. To determine this effect, mycelia were grown in YEPD medium for 12, 24, 36 and 48 h before enzyme treatment. The results showed that mycelia at the age of 24 h produced the largest amount of protoplasts (Fig. 1e) with the best regeneration frequency (Fig. 1f). Additionally, the optimal concentrations of polyethylene glycol (PEG) and Ca^2+^ for protoplast transformation were also investigated using the plasmid pBSKII-*toCas9*-*hph* containing the hygromycin resistance marker. We observed that PEG had a significant effect on transformation efficiency with the best efficiency achieved at 60% (Fig. 1g), whereas the concentration of Ca^2+^ had less effect (Fig. 1h). Based on these results, we established the optimal conditions for protoplast preparation by treatment of 24 h-old mycelia with Yatalase for 1 h, and the conditions for PEG-mediated transformation by using 60% PEG and 50 mM Ca^2+^.

### Establishment of the Cas9 stable expression *Nodulisporium* sp. (No. 65-12-7-1)

**Figure 2:**
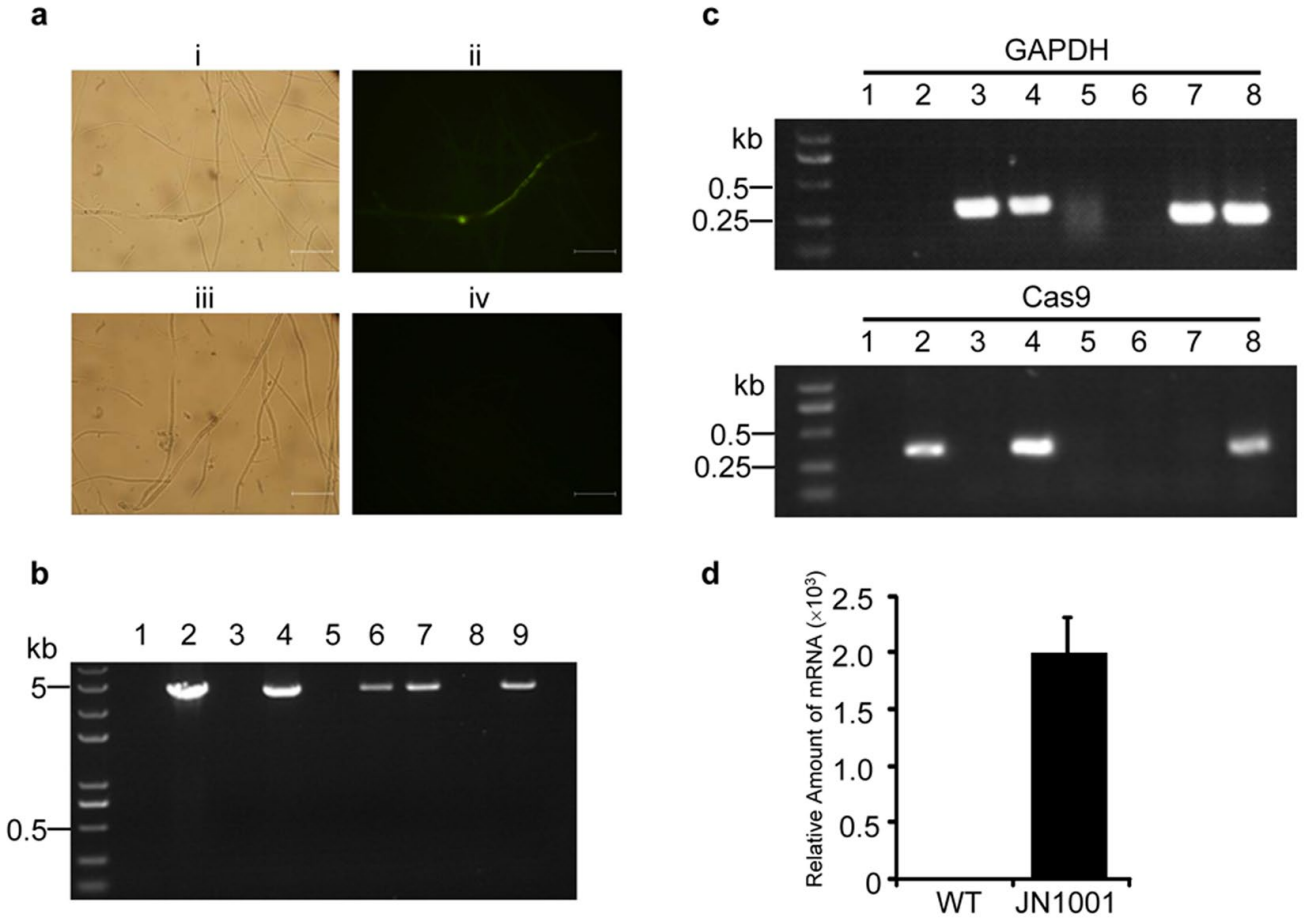
Establishment of the Cas9 stable expression *Nodulisporium sp*. (No. 65-12-7-1). (**a**) Analysis of the Cas9-eGFP expression in the mycelia transfected with pDHt/sk-Ppdc-*toCas9*-*eGFP*-Tpdc (i, ii) and wild-type (iii, iv). The bar represents 100 μm. (**b**) Analysis of the integration of *cas9* gene into the genome. (lane 1: wild-type; lane 2: pBSKII-*tocas9*-*hph*; lane 3: JN1002; lane 4: JN1001; lane 5: JN1003; lane 6: JN1004; lane 7: JN1005; lane 8: JN1006; lane 9: JN1007). (**c**) RT-PCR analysis of the Cas9 expression. Upper panel for GAPDH and lower panel for Cas9. lane 1: H_2_O; lane 2: pBSKII-*toCas9*-*hph*; lane 3: genomic DNA of wild-type; lane 4: genomic DNA of JN1001; lane 5: RNA of wild-type; lane 6: RNA of JN1001; lane 7: cDNA of wild-type; lane 8: cDNA of JN1001. (**d**) Real-time PCR quantitative analysis of Cas9 expression in WT and JN1001.

Since we had established the protoplast isolation and transformation system for *Nodulisporium* sp. (No. 65-12-7-1), we then aimed to build an efficient CRISPR-Cas9 system in this fungus. As the *Nodulisporium* codon-optimized *cas9* is unavailable due to the lack of codon usage information in this genus, we first investigated whether the *T*. *reesei* codon-optimized *cas9* (*toCas9*) could express in *Nodulisporium* sp. (No. 65-12-7-1) by transformation of the fungus with the Cas9-eGFP expressing vector pDHt/sk-Ppdc-*toCas9*-*eGFP*-Tpdc. We observed an evident fluorescence in the transformant (Fig. 2a, ii), but not in the control strain (Fig. 2a, iv), suggesting that *toCas9* could also function in *Nodulisporium* sp. (No. 65-12-7-1). Then, a plasmid pBSKII-*toCas9*-*hph* containing the *cas9* cassette under the control of *A*. *nidulans trpC* promoter and terminator was constructed and transformed into *Nodulisporium* sp. (No. 65-12-7-1) according to the established protoplast transformation procedure. Seven hygromycin-resistance fungus clones were randomly selected. Since the plasmid pBSKII-*toCas9*-*hph* we used for Cas9 expression does not contain a fungus origin of replication, integration of the plasmid into the genome is required for the growth of transformants in the presence of hygromycin. As expected, the integration of hygromycin resistance gene into the genomes of all seven clones was confirmed (Supplementary Fig. S1). However, since the integration of plasmid into the genome is a random event, cleavage in the *cas9* coding region during integration may occur. To select the clones in which the *cas9* gene was correctly inserted into their genomes, genomic PCR using primers flanking the *cas9* coding sequence was performed, and four of the seven clones were confirmed to have the *cas9* gene correctly integrated into their genomes (Fig. 2b, lane 4, 6, 7, 9). We then selected one clone (JN1001) for the Cas9 expression assay. To investigate the expression of Cas9 in JN1001, total RNA from JN1001 and the wild-type strain was extracted and subjected to reverse transcription-PCR analysis (RT-PCR) using glyceraldehyde-3-phosphate dehydrogenase (GAPDH) (Accession number KY977747) as a reference. As the primers used to amplify GAPDH flank an intron, a smaller amplicon should be generated from cDNA (Fig. 2c, upper panel, lane 7, 8) compared to that from genomic DNA (Fig. 2c, upper panel, lane 3, 4). The complete disappearance of the genomic DNA-derived band in RT-PCR indicated that the genomic DNA was completely elminated in the RNA samples and the amplicons generated by RT-PCR were exclusively from mRNA, which was also confirmed by direct PCR amplication of the RNA samples without RT (Fig. 2c, upper panel, lane 5 and lane 6). Under these circumstances, Cas9 expression was only detected in JN1001 (Fig. 2c, lower panel, lane 8), but not in the wild-type (Fig. 2c, lower panel, lane 7). Real-time quantitative PCR analysis also revealed expression of Cas9 in JN1001, but not in wild-type (Fig. 2d). These results indicated that a stable Cas9 expression strain was established. Moreover, we confirmed that JN1001 produced similar amount of demethoxyviridins to that of wild-type (Supplementary Fig. S2), suggesting that the expression of Cas9 had less effects on the production of secondary metabolites and the JN1001 strain was suitable for studing the biosynthesis of demethoxyviridin.

### CRISPR-Cas9-based gene disruption by *U6* promoter driving gRNA expression

The CRISPR-Cas9 system requires both the enzyme Cas9 and an engineered gRNA whose expression is usually under the control of RNA polymerase III promoters (RNAPIII) such as the *U6* small nuclear RNA (*U6* snRNA) promoter. In the genome of *Nodulisporium* sp. (No. 65-12-7-1), one homologue of the mammalian *U6* snRNA gene was identified by local blast according to the method described previously^10^. Sequence alignment of this gene with human *U6* snRNA and those identified in *P*. *oryzae* and *A*. *fumigatus* af293 indicated that the *U6* snRNA gene of *Nodulisporium* sp. was highly similar to those found in fungi (Supplementary Fig. S3). The 500 bp upstream of the *U6* snRNA gene was then used as the *U6* promoter (*U6*_Nod_) to express gRNA in *Nodulisporium* sp. (No. 65-12-7-1). To test the feasibility of the CRISPR-Cas9, we randomly selected the P450 gene *g3279* (Accession number KY930910) from the genome of *Nodulisporium* sp. (No. 65-12-7-1) as the mutagenesis target. The gRNA sequence containing the 20-nt target of *g3279* was designed using a online web tool (http://zifit.partners.org/ZiFiT/ChoiceMenu.aspx) and reffered to as gRNA-*g3279*. Then the gRNA-*g3279* expressing plasmid pBSKII-PtrPC-*neo*-TtrPC-*U6*_Nod_-gRNA-*g3279* was transformed into the Cas9-expressing strain JN1001. Ten transformants growing on G418-containing medium were randomly picked out and their genomic DNA was purified. Sucessful incorporation of the gRNA-expressing vector in all these transformants was confirmed by PCR amplification of the *neo* gene from their genomic DNA (Supplementary Fig. S4a). Then we performed genotyping PCR to check the indels of *g3279* using primers flanking the targeting site, and found that all the clones gave identical PCR products to wild-type (Supplementary Fig. S4b). Sequencing of these PCR products revealed that in all ten transformants no mutations around the target site were observed. The low efficiency of gene mutation was possibly caused by the inactivation of the endogenous *U6* promoter. To address this issue, we constructed a plasmid pBSKII-PtrPC-*neo*-TtrPC-*U6*_Asp_-gRNA-*g3279* to express gRNA-*g3279* using the *U6* promoter derived from *A*. *oryzae* that has been shown to be active in *A*. *oryzae* RIB40^12^. As a result, only two of the twelve selected transformants (clone 3 and clone 8) were found to have mutations at the target sites (Supplementary Fig. S4c and d). The above results indicated that though the *U6* promoter-driving CRISPR-Cas9 system was able to induce gene mutagenesis, the mutation efficiency was too low to be practical.

### An efficient CRISPR-Cas9-based gene disruption system by co-transformation of *in vitro* transcriptional gRNA and linear marker gene cassettes

**Figure 3:**
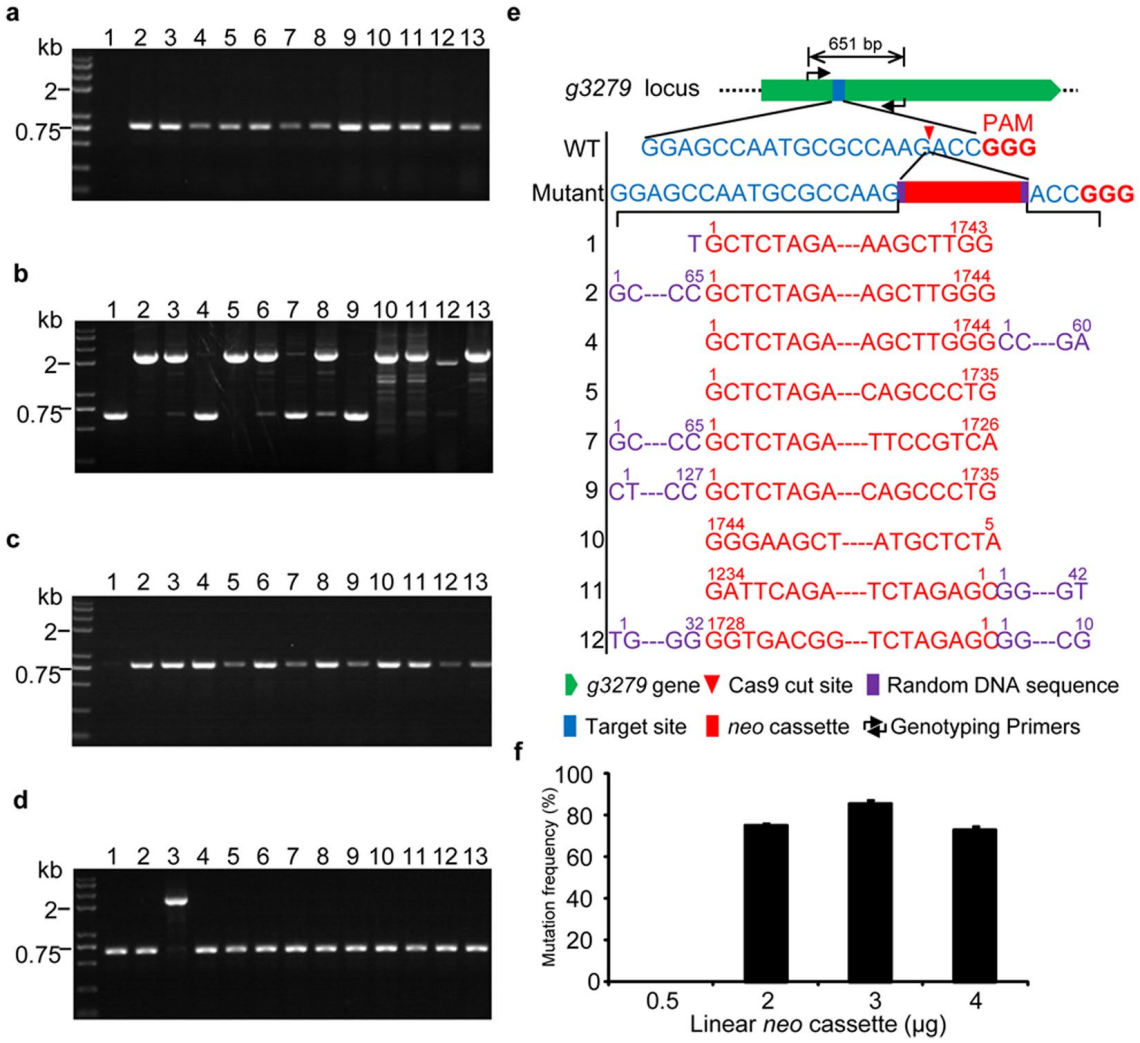
Efficient gene disruption by co-transformation of *in vitro* transcriptional gRNA and the linear marker gene cassette. (**a**) Analysis of the integration of *neo* gene into the genome of G418-resistance clones generated by co-transformation of the *in vitro* transcriptional gRNA and linear *neo* cassette into the JN1001. (**b**) PCR amplification of the DNA regions surrounding the target site of the clones described in (**a**) using primers flanking the target site. (**c**) Analysis of the integration of *neo* cassette into the genome of G418-resistance clones generated by co-transformation of the *in vitro* transcriptional gRNA and circular plasmid pBSKII-PtrPC-*neo*-TtrPC containing the *neo* cassette into the JN1001. (**d**) PCR amplification of the DNA regions surrounding the target site of the clones described in (**c**) using primers flanking the target site. (**e**) Sequence analysis of PCR products generated in (**b**). (**f**) Analysis of the effects of usage amount of linear *neo* cassette on the mutation efficiency. ((**a–d**), lane 1: JN1001; lane 2-13: G418-resistance clones (No. 1-12)).

Considering the low efficiency of gene mutation using *U6* promoter-driven expression of gRNA, we decided to deliver gRNA directly into the Cas9-expressing fungus JN1001 after *in vitro* transcription. To select the transformants into which the gRNA was successfully incorporated, the linear *neo* cassette was simultaneously transformed. Twelve clones growing on the G418 plates were picked and subjected to DNA extraction for target site sequencing. The successful transformation of gRNA into the fungi was confirmed by PCR ampilification of the *neo* gene from the genomic DNA of all twelve clones (Fig. 3a). Next, the DNA regions surrounding the target site were amplified using primers flanking the target site. Unexpectedly, nine of the twelve transformants yielded amplicons with molecular weights between 2 kb and 3 kb, which were much larger than that of wild type (Fig. 3b), suggesting some DNA fragments might be inserted in the target region. To examine the mutagenesis in further detail, the unexpectedly sized PCR products from nine clones and those possessing the expected size from four clones were extracted from the gel and subjected to sequencing. In all of the nine clones that showed abnormal bands, a *neo* cassette with slightly modified ends by either nucleotide truncation or random sequence attachment was inserted to the cleavage site of Cas9 at 3 bp upstream of protospacer adjacent motif (PAM) (Fig. 3e and Supplementary Table S3). In contrast, in the four clones that exhibited normal bands, neither insertion nor deletion in the target sites was detected. These results raised the possibility that the linear *neo* cassette not only accounted for the selection of transformants, but also functioned to improve the mutation frequency by insertion of itself at the break site. To verify our hypothesis, we used a circular vector containing the same *neo* cassette to replace the linear *neo* cassette fragment for transformation. After confirming that all the twelve transformants had the *neo* gene integrated into their genomes (Fig. 3c), the target sites were amplified and sequenced. Only one of the twelve selected transformants yielded an amplicon with large size when transformed with circular plasmids (Fig. 3d), which was consistent with our expectations. Sequencing of the PCR products from all these clones revealed that only the clone showing larger band was mutated by insertion of a portion of the transforming plasmid into the target site, none of the other clones was mutated around the target site. For further confirmation, two independent transformations using the linear *neo* cassette and circular plasmid were carried out to yield a total of 41 and 22 G418-resistance clones, respectively. Remarkably, 28 of 41 linear *neo* cassette transformants (68.3%) were mutated, whereas only one of the 22 circular plasmid clones was mutated (Table 1). To verify that the above mentioned gene disruption is really a CRISPR-Cas9-mediated process, transformation of the wild-type strain or JN1001 with either the linear *neo* cassette or circular plasmid was performed. Ten clones in each transformation were randomly selected, and all these clones gave same-sized amplicons to that of wild-type (Supplementary Fig. S5). We sequenced these PCR products and did not observe any mutations or insertions around the target sites in all these clones. These results clearly indicated that simultaneous delivery of the linear marker gene cassette with *in vitro* transcriptional gRNA was able to significantly improve the mutation frequency and this could be useful for gene disruption in those fungal strains in which the common CRISPR-Cas9 system was not efficient.

**Table 1:** Comparison of the mutation rate in transformates generated by linear *neo* cassette and circular plasmid.

	clones tested	mutation clones	Mutation rate (%)
linear *neo* cassette + gRNA	41	28	68.3
pBSKII-PtrPC-*neo*-TtrPC + gRNA	22	1	4.5

Since we had highlighted the role of linear *neo* cassette in gene disruption, we then turned our attention to determining the optimal amount to achieve the maximal mutation frequency. As shown in Fig. 3f and Supplementary Fig. S6, we found that 2 μg of linear *neo* cassette (the molar ratio of gRNA to linear *neo* cassette was approximately 180: 1) was sufficient to achieve a high mutation frequency, and 3 μg reached the maximum, and more than 3 μg would have deleterious effects.

### The newly established CRISPR-Cas9 system functions well in other phylogenetically distinct fungi

**Figure 4:**
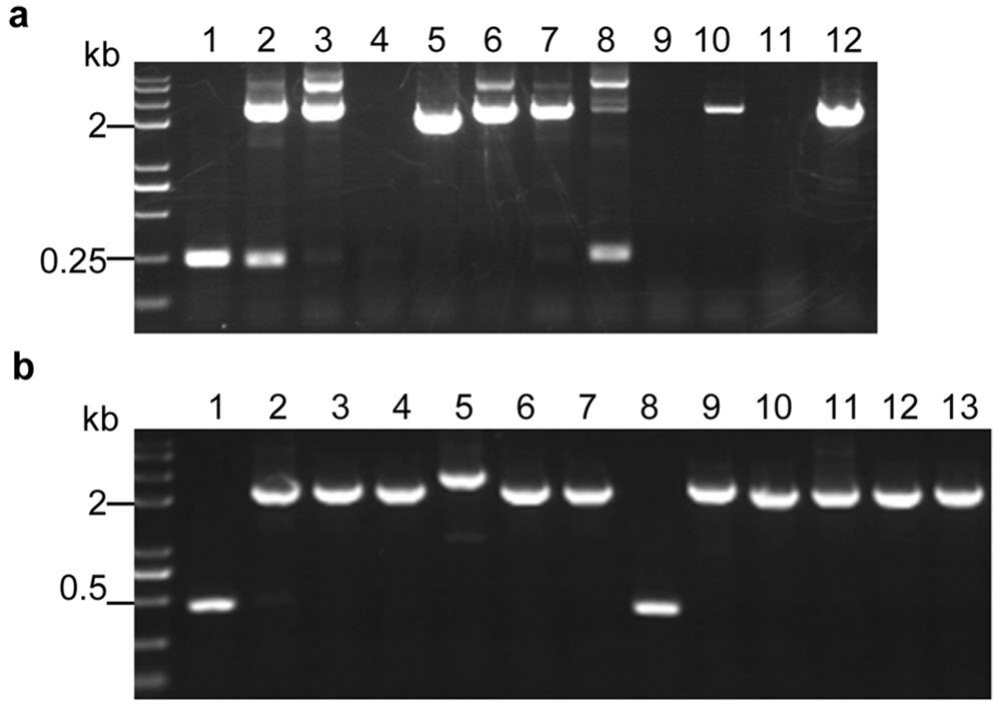
The newly established CRISPR-Cas9 system functions well in *A*. *oryzae* NSAR1 and *S*. *minima* (No. 40-1-4-1). (**a**) Targeted disruption of *wA* in *A*. *oryzae*. NSAR1 (1: JA1001; lane 2-12: *argB*-resistance clones (No. 1-11)). (**b**) Targeted disruption of *HdaA* in *S*. *minima* (No. 40-1-4-1). (1: JS1001; lane 2-13: G418-resistance clones (No. 1-12)).

To investigate whether the above established CRISPR-Cas9 system through co-transformation of linear marker gene cassette and *in vitro* transcriptional gRNA can be applied to other filamentous fungi, we tested it in two other phylogenetically distinct fungal strains *A*. *oryzae* NSAR1 and *S*. *minima* (No. 40-1-4-1), which are from two different classes of the phylum Ascomycota. Recently, the CRISPR-Cas9 system has been successfully applied to *A*. *oryzae* RIB40 by transformation with a single vector pUNAFNC9gwA1 containing both the *cas9* cassette and gRNA-*wA* cassette^12^. Here, we used our strategy to target the same gene *wA* (AO090102000545) in *A*. *oryzae* NSAR1. The *in vitro* transcriptional gRNA-*wA* and the linear *argB* cassette that was amplified from pTAex3 using primers *argB*-F and *argB*-R were co-transformed into the Cas9-expressing *A*. *oryzae* NSAR1 (JA1001). Eleven clones growing on L-arginine deficient medium were randomly selected for genomic DNA extraction. Diagnostic PCR used primers flanking the target site revealed that eight of the eleven transformants gave abnormal large amplicons (Fig. 4a), and this was shown by PCR product sequencing to be caused by insertion of the linear *argB* cassette at the cut site. For further confirmation, we tested our system in another seldom studied fungus *S*. *minima* (No. 40-1-4-1) by targeting the histone deacetylase A (*HdaA*) gene (Accession number KY930909). After co-transformaiton of gRNA-*HdaA* and the linear *neo* cassette to the Cas9-expressing *S*. *minima* (JS1001), twelve transformants were randomly selected for genomic DNA extraction. Similarly with those found in *Nodulisprium* sp. and *A*. *oryzae* NSAR1, we observed that eleven of twelve selected transformants were mutated by insertion of linear *neo* cassette (Fig. 4b). These results clearly demonstrated the versatility of our established CRISPR-Cas9 system.

## Discussion

CRISPR-Cas9-based gene editing has become one of the most important technologies in life science due to its unprecedented control over genomes from animals, plants, fungi, bacteria and many other organisms. As compared to its use in mammalian systems, the application of CRISPR-Cas9 system in filamentous fungi is still in a preliminary state. There are a few recent reports regarding the use of the CRISPR-Cas9 system for performing loss-of-function studies in filamentous fungi. Most of these studies adopted an approach to transform fungal strains with a solo vector containing both the gRNA cassette under the control of *U6* promoter and *cas9* cassette driven by a constitutive promoter. Gene mutagenesis was achieved during repair of the DSBs induced by CRISPR-Cas9 through either NHEJ or HDR^10, 12, 15, 17, 26^. However, some researchers preferred to establish a Cas9 stable expression strain, for use as a recipient for the subsequent gRNA transformation in a separate plasmid^11, 14, 27^. In this way, the Cas9-expressing fungus may serve as a universal strain for CRISPR-Cas9-mediated mutagenesis. In some cases, due to the lack of confirmed RNA polymerase-III-based promoters, direct transformation with *in vitro* transcriptional gRNA was also carried out^14^. Unlike all of the above-mentioned approaches, we have developed a highly efficient and versatile CRISPR-Cas9-based gene disruption strategy by simultaneous transformation of the *in vitro* transcriptional gRNA and the linear selectable marker gene cassette into a Cas9-expressing strain. The utility of this system has been demonstrated in three phylogenetically distinct fungal strains *Nodulisporium* sp. (No. 65-12-7-1), *A*. *oryzae* NSAR1, and *S*. *minima* (No. 40-1-4-1). In addition, we have demonstrated that this system also worked well in two other fungal strains from *Talaromyces* (Li, S. Y., Hu, D. unpublished data) and *Myrothecium*, (Lin, F. L., Hu, D. unpublished data). As compared to previous reports on the application of the CRISPR-Cas9 system in filamentous fungi, our system might have three major advantages: (i) the use of *in vitro* transcriptional gRNA which does not rely on the endogenous *U6* promoter in individual fungus strains, improving the versatility of the present system, (ii) the linear maker gene cassette not only allows for the selection of transformants, but also functions to improve the gene mutation frequency, which is especially useful for gene targeting in the strains with low efficiency of CRISPR-Cas9 system. (iii) the insertion of the maker gene cassette into the cut site is useful for screening of mutant strains by PCR, which show larger amplicons than that of the wild type.

Analysis of the DNA fragments inserted at the Cas9 cut site indicated that they were mainly derived from the linear *neo* cassette, but some of them were flanked by unknown sequences (Fig. 3e and Supplementary Table S3). To investigate the origin of these flanking sequences, we have performed extensive blast in NCBI database. We found that they appear to correspond to the sequences in shuttle vector database ((taxid:29278) at NCBI), especially the T3 promoter sequence, which is not contained in the linear *neo* cassette. It would be very interesting to demonstrate whether these sequences function during genome integration of the linear *neo* cassette at the Cas9 cut site.

One of the major findings in our present data is that using the linear marker gene cassette produces a much higher mutation frequency than using circular plasmid. Indeed, integration of transforming DNA at the Cas9 cut site has been described in a mammalian cell CRISPR system^28^. Recently, Fuller *et al*. also observed a high frequency integration of linearized plasmid or transforming PCR fragments at the Cas9 cut site in *A*. *fumigatus*, Af293 and CEA10^11^. Interestingly, a very close study conducted by Zhang *et al*. showed that insertion of the linear transforming fragment at the target site was not observed when transforming of a NHEJ-deficient *A*. *fumigatus* strain^27^. These results suggested that NHEJ might be responsible for the insertion of linear maker gene cassette into the Cas9 cut site. In *Nodulisporium* sp. (No. 65-12-7-1), we did not observe any mutations caused by NHEJ-mediated single nucleotide insertion/deletion in the transformants, although this has often been reported in the literature. Considering the high frequency of insertion of the linear selectable marker gene cassette into the Cas9 cut site, we could rule out the possibility of insufficient Cas9 expression. We thus proposed that an accurate NHEJ repair of the DSB induced by CRISPR-Cas9 might account for the low efficiency of the NHEJ-mediated nucleotide deletion/insertion. However, this scenario would be disrupted by the presence of a linear DNA fragment that recruited NHEJ proteins to facilitate integration of itself into the Cas9 cut site, leading to a high frequency of mutagenesis.

## Materials and Methods

### Strains, plasmids, culture conditions, antibiotics

All strains and plasmids used in this study are listed in Supplementary Table S1. *Escherichia coli* strain DH5α used as the general cloning host were cultured in Luria Broth (LB) liquid medium (1% [w/v] tryptone, 0.5% [w/v] yeast extract, 1% [w/v] NaCl) or on LB agar plates at 37 °C. The three fungal strains *Nodulisporium* sp. (No. 65-12-7-1), *A*. *oryzae* NSAR1 and *S*. *minima* (No. 40-1-4-1) were grown on yeast extract peptone dextrose (YEPD) medium (2% [w/v] dextrin, 1% [w/v] tryptone, 0.5% [w/v] yeast extract, 0.05% [w/v] MgSO_4_·7H_2_O, 0.5% [w/v] KH_2_PO_4_) at 28 °C for genomic DNA extraction or protoplast preparation. Transformants were selected on M agar medium (0.2% [w/v] NH_4_Cl, 0.1% [w/v] (NH_4_)_2_SO_4_, 0.05% [w/v] KCl, 0.05% [w/v] NaCl, 0.1% [w/v] KH_2_PO_4_, 0.05% [w/v] MgSO_4_·7H_2_O, 0.002% [w/v] FeSO_4_·7H_2_O, 2% [w/v] glucose, 1.2 M sorbitol, 0.8% agar or 1.5% agar, pH 5.5) containing corresponding antibiotics. Plasmids pDHt/sk-Ppdc-*toCas9*-Tpdc and pDHt/sk-Ppdc-*toCas9*-*eGFP*-Tpdc, both of which contain the *Trichoderma reesei* codon-optimized *cas9* (*toCas9*) and hygromycin B phosphotransferase (*hph*) coding sequences, were kindly provided by Prof. Zhihua Zhou from CAS-Key Laboratory of Synthetic Biology, Shanghai, China^14^. These two vectors were used to construct the Cas9-expressing vector pBSKII-*toCas9*-*hph* used in this study. The pUCm-T vector (Sangon Biotech Co., Ltd. Shanghai, China) was used to maintain gRNA expression templates. pUNAFNC9gwA1 containing the *A*. *oryzae* codon-optimized *cas9* gene cassette, which was kindly provided by Prof. Maruyam from the University of Tokyo^12^, was used to construct Cas9-expressing vector pAdeA-*cas9* in *A*. *oryzae* NSAR1. Antibiotics were added to the above mediums as required at a concentration of 50 μg/mL kanamycin sulfate, 100 μg/mL ampicillin sodium salt. For *Nodulisporium* sp. (No. 65-12-7-1), 100 μg/mL hygromycin B and 200 μg/mL G418 were used, while for *S*. *minima* (No. 40-1-4-1), 350 μg/mL hygromycin B and 300 μg/mL G418 were used.

### DNA manipulations

Primer synthesis and DNA sequencing were performed by Sangon Biotech Co., Ltd. (Shanghai, China) (Supplementary Table S2). Plasmid extraction and DNA purification were carried out with commercial kits (Sangon Biotech Co., Ltd. Shanghai, China). Restriction enzymes and other DNA modification reagents were purchased from Thermo Fisher Scientific (Shenzhen, China) and TOYOBO Co., Ltd. (Osaka, Japan). PCR amplification was carried out on a Mastercycler nexus gradient (Eppendorf, Hamburg, Germany) with either Taq DNA polymerase (TaKaRa, Dalian, China) or KOD FX DNA polymerase, KOD Plus DNA polymerase (TOYOBO, Osaka, Japan).

### Genomic DNA extraction

Filamentous fungi were grown in 10 mL YEPD liquid medium at 28 °C for 48 h. Mycelia were harvested and washed with distilled water. After that, they were ground in liquid nitrogen and suspended in 500 µL 2 × CTAB buffer (100 mM Tris-HCl, 1.4 M NaCl, 20 mM EDTA, pH 8.0) with shaking at 60 °C for 30 min. The genomic DNA was purified by phenol/chloroform extraction solution followed by isopropanol precipitation. The purified DNA was dried and dissolved in ddH_2_O.

### Construction of Cas9 expression vectors

The *A*. *nidulans trpC* promoter (PtrPC) and terminator (TtrPC) were amplified from pDHt/sk-Ppdc-*toCas9*-*eGFP*-Tpdc using two primer pairs PtrpC-XbaІ-F/PtrpC-R and TtrpC-EcoRV-F/TtrpC-HindШ-R, respectively (The primer sequences were summarized in Supplementary Table S2). The resulting two PCR fragments were combined by overlap PCR and then ligated into the XbaI/HindIII sites of pBluescript SKII to generate pBSKII-PtrPC-EcoRV-TtrPC. The *toCas9* coding sequence was amplified from pDHt/sk-Ppdc-*toCas9*-Tpdc using primers Flag-*cas9*-F and *cas9*-R and inserted into the EcoRV site of pBSKII-PtrPC-EcoRV-TtrPC to give pBSKII-PtrPC-Flag-*toCas9*-TtrPC. The *hph* cassette was amplified from pDHt/sk-Ppdc-*toCas9*-*eGFP*-Tpdc using primers PtrpC-AanІ-F and TtrpC-AanІ-R, and ligated into the AanІ site of the pBSKII-PtrPC-Flag-*toCas9*-TtrPC to generate pBSKII-*toCas9*-*hph*, which was used to express Cas9 in *Nodulisporium* sp. (No. 65-12-7-1) and *S*. *minima* (No. 40-1-4-1). For expressing Cas9 in *A*. *oryzae* NSAR1, the *cas9* cassette was amplified from pUNAFNC9gwA1 using primers *amy*-F/*amy*-R, and then fused into the XbaІ site of pAdeA to generate pAdeA-*cas9* using In-Fusion HD Cloning Kits (Takara).

### Construction of gRNA vectors for expression in *Nodulisporium* sp. (No. 65-12-7-1)

The coding sequence for the neomycin phosphotransferase gene (*neo*) was amplified from pcDNA3.1 using primers *neo*-F/*neo*-R, and then ligated into the EcoRV site of pBSKII-PtrPC-EcoRV-TtrPC to generate pBSKII-PtrPC-*neo*-TtrPC. The *U6* promoter from *Nodulisporium* sp. (No. 65-12-7-1) (*U6*_Nod_) was amplified from its genomic DNA using primers *U6*p-F1/*U6*p-R1, while the *U6* promoter from *A*. *oryzae* (*U6*_Asp_) was amplified from pUNAFNC9gwA1 using *U6*p-AanI-F2/*U6*p-R2. The fragment containing *g3279-*targeting protospacer, gRNA scaffold and *U6* terminator was prepared by PCR amplification using pUNAFNC9gwA1 as template with primers gRNA-*U6*t-F/*U6*t-AanI-R, which was then fused with the above *U6* promoters by overlap PCR and inserted into the AanI site of pBSKII-PtrPC-*neo*-TtrPC to yield pBSKII-PtrPC-*neo*-TtrPC-*U6*_Nod_-gRNA-*g3279* and pBSKII-PtrPC-*neo*-TtrPC-*U6*_Asp_-gRNA-*g3279* for expressing gRNA in *Nodulisporium* sp. (No. 65-12-7-1). The sequences of these DNA elements used for vector construction are shown in supplementary sequence data.

### Preparation of *in vitro* transcriptional gRNA

*In vitro* transcription of gRNA was carried out as previously described^14^ with slight modifications. Briefly, the synthesized gRNA scaffold fused with *eGFP* was ligated in pUCm-T to generate pUCm-gRNAscaffold-*eGFP*. The gRNA cassettes containing T7 promoter, protospacer sequence and synthetic gRNA scaffold for targeting *g3279*, *wA* and *HdaA* were PCR amplifified from pUCm-gRNAscaffold-*eGFP* using three primer pairs gRNA-*g3279*-F/*eGFP*-R, gRNA-*wA*-F/*eGFP*-R and gRNA-*HdaA*-F/*eGFP*-R, and inserted in pUCm-T to give pUCm-gRNA-*g3279*, pUCm-gRNA-*wA*, pUCm-gRNA-*HdaA*, respectively. These vectors were used as template for PCR amplification with primers pUCm-F/gRNA-R, the resulting PCR products were utilized for *in vitro* transcriptional of gRNA using the T7 RiboMAX^TM^ Express Large Scale RNA Production System (Promega, China).

### Preparation of the linear marker gene cassette

The linear *neo* maker gene cassette was amplified from pBSKII-PtrPC-*neo*-TtrPC using primers PtrpC-XbaІ-F/TtrpC-HindШ-R. The linear *argB* maker genea cassette was amplified from pTAex3 using primers *argB*-F/*argB*-R.

### Protoplast preparation, transformation and strain validation by genotyping PCR

Preparation and transformation of *Nodulisporium* sp. (No. 65-12-7-1) protoplast were performed according to a modified version of the method of Kitamoto^29^. Briefly, *Nodulisporium* sp. (No. 65-12-7-1) was inoculated in 100 mL YEPD liquid medium and shaken for 24 h at 28 °C. The mycelia were then collected by filtration, and washed with sterilized distilled water. After that, the mycelia were suspended in 10 mL enzymatic solution and incubated at 30 °C for 1 h by mild agitation (50 strokes/min). Protoplast release was checked by microscopic observation. The protoplasts were then separated from mycelia by filtration through a sterilized cotton-packed injector and diluted with an equal volume of TF Solution II (1.2 M sorbitol, 50 mM CaCl_2_·2H_2_O, 35 mM NaCl, 10 mM Tris-HCl, pH 7.5). Then they were gently precipitated by centrifugation and washed once again with 10 mL TF Solution II. Finally, the protoplasts were resuspended in TF Solution II to a concentration of 2 × 10^7^/mL for subsequent transformation.

About 5-10 μg of plasmids, DNA fragment or *in vitro* transcriptional gRNA was added into 200 μL of the above protoplast suspension and incubated on ice for 30 min. The resulting DNA-protoplast mixture was serially mixed with 250, 250, and 850 μL of TF Solution III (60% PEG 4000, 50 mM CaCl_2_·2H_2_O, 10 mM Tris-HCl, pH 7.5) and kept at room temperature for 20 min. The PEG-treated protoplast suspension was diluted with 5 mL TF Solution II, and centrifuged at 420 × *g* for 10 min at 4 °C. After that, the protoplasts were resuspended in 200 μL of TF Solution II and mixed with 5 mL of 0.8% agar M medium containing hygromycin B. The resulting mixture was then poured onto M agar medium containing 1.5% agar and hygromycin B. After 7 days cultivation at 28 °C, transformants were inoculated onto a new M agar plate (without sorbitol). About 12 colonies were randomly picked and cultured in YEPD for genomic DNA extraction. Gene disruption in individual clones was validated by sequence of the PCR products amplified with primer pairs (*g3279*-F/*g3279*-R, *wA*-F/*wA*-R, *HdaA*-F/*HdaA*-R) flanking the target site. Preparation and transformation of protoplasts of *A*. *oryzae* NSAR1 were carried out according to the method of Kitamoto^29^. The preparation and transformation of *S*. *minima* (No. 40-1-4-1) protoplasts were recently established in our lab (Zhang, J. W., Hu, D. unpublished data).

### Accession numbers

The nucleotide sequences of *g3279* and GAPDH from *Nodulisporium* sp. (No. 65-12-7-1) and the *HdaA* gene from *S*. *minima* (No. 40-1-4-1) reported in this study have been deposited into Genbank under accession No. KY930910, KY977747 and KY930909, respectively.

### Data Availability

All data generated or analyzed during this study are included in this published article (and its Supplementary Information files).

## Electronic supplementary material

supplementary information
