# Supplementary material for: Development of a versatile and conventional technique for gene disruption in filamentous fungi based on CRISPR-Cas9 technology
Source: Sci Rep. 2017 Aug 23;7:9250. doi: 10.1038/s41598-017-10052-3 (PMC5569088; doi:10.1038/s41598-017-10052-3)
Supplement: Supplementary file 1 — supplementary information [file 41598_2017_10052_MOESM1_ESM.pdf]

Development of a versatile and conventional technique for gene disruption in filamentous fungi based on CRISPR-Cas9 technology

Yan-Mei Zheng,[1,+] Fu-Long Lin,[1,+] Hao Gao,[1,*] Gen Zou,[2] Jiang-Wei Zhang,[1] Gao-Qian Wang,[1] Guo-Dong Chen,[1] Zhi-Hua Zhou,[2] Xin-Sheng Yao,[1] Dan Hu[1,3,*]

[1]Institute of Traditional Chinese Medicine and Natural Products, College of Pharmacy / Guangdong Province Key Laboratory of Pharmacodynamic Constituents of TCM and New Drugs Research, Jinan University, Guangzhou, 510632, China

[2]CAS-Key Laboratory of Synthetic Biology, Institute of Plant Physiology and Ecology, Shanghai Institutes for Biological Sciences, Chinese Academy of Sciences, Shanghai, 200000, China

[3]State Key Laboratory of Bioorganic and Natural Products Chemistry, Shanghai Institute of Organic Chemistry, University of Chinese Academy of Sciences, Shanghai, 200000, China

[+]these authors contributed equally to this work

*Address correspondence to Dan Hu, thudan@jnu.edu.cn or Hao Gao, tghao@jnu.edu.cn

Supplemental Information

1. Supplementary Sequence Data

Sequence data S1: Nucleotide sequence of Flag-tagged *toCas9*

Sequence data S2: Nucleotide sequence of PtrPC-*neo*-TtrPC cassette

Sequence data S3: Nucleotide sequence of *U6* promoter of *Aspergillus oryzae* RIB40

Sequence data S4: Nucleotide sequence of *U6* promoter of *Nodulisporium* sp. (No. 65-12-7-1)

Sequence data S5: Nucleotide sequence of *U6* terminator

Sequence data S6: Nucleotide sequence of gRNA-*g3279* for *U6* promoter-mediated *in vivo* transcription

Sequence data S7: Nucleotide sequence of gRNA-*g3279* for *T7* promoter-mediated *in vitro* transcription

Sequence data S8: Nucleotide sequence of gRNA-*wA* for *T7* promoter-mediated *in vitro* transcription

Sequence data S9: Nucleotide sequence of gRNA-*HdaA* for *T7* promoter-mediated *in vitro* transcription

2. Supplementary Tables

Table S1. Strains and plasmids used in this study

Table S2. Primers used in this study

Table S3. Nucleotide sequences of the random DNA fragment attached to the *neo* cassette in *g3279* mutants in Fig. 3e

3. Supplementary Figures

Fig. S1. Analysis of the integration of *hph* gene into the genome of the clones transformed with pBSKII-*tocas9-hph*.

Fig. S2. HPLC analysis of the secondary metabolites of wild-type and JN1001.

Fig. S3. Sequence alignment of *U6* snRNA genes.

Fig. S4. CRISPR-Cas9-based gene disruption by *U6* promoter driving gRNA expression.

Fig. S5. Both Cas9 and *in vitro* gRNA are essential for mutations.

Fig. S6. Analysis of the effects of usage amount of linear *neo* cassette on the mutation

efficiency.

Sequence data S1: Nucleotide sequence of Flag-tagged *toCas9*

ATGgattacaaggatgacgacgataagCCCAAGAAGAAGCGCAAGGTCGACAAGAAGTACAGC
ATTGGCCTGGACATTGGCACGAACTCGGTCGGCTGGGCCGTCATCACGGACGAGT
ACAAGGTCCCCTCCAAGAAGTTTAAGGTCCTGGGCAACACCGACCGCCACTCCAT
CAAGAAGAACCTCATTGGCGCCCTGCTCTTCGACTCCGGCGAGACCGCCGAGGCC
ACCCGCCTCAAGCGCACCGCCCGCCGCCGATACACGCGCCGCAAGAACCGCATCT
GCTACCTGCAGGAGATTTTCTCCAACGAGATGGCCAAGGTCGACGACTCCTTCTTT
CACCGCCTGGAGGAGTCGTTCCTCGTCGAGGAAGACAAGAAGCACGAGCGCCAC
CCCATCTTTGGCAACATTGTCGACGAGGTCGCCTACCACGAGAAGTACCCCACGAT
CTACCACCTGCGCAAGAAGCTCGTCGACTCCACCGACAAGGCCGACCTCCGCCTG
ATCTACCTCGCCCTGGCCCACATGATTAAGTTCCGCGGCCACTTTCTGATCGAGGG
CGACCTCAACCCCGACAACAGCGACGTCGACAAGCTGTTCATCCAGCTCGTCCAG
ACCTACAACCAGCTCTTTGAGGAGAACCCCATTAACGCCTCCGGCGTCGACGCCA
AGGCCATCCTCTCGGCCCGCCTCTCCAAGAGCCGCCGACTCGAGAACCTGATCGC
CCAGCTGCCCGGCGAGAAGAAGAACGGCCTGTTCGGCAACCTCATCGCCCTCTCC
CTGGGCCTCACCCCCAACTTCAAGTCGAACTTTGACCTCGCCGAGGACGCCAAGC
TGCAGCTCTCCAAGGACACCTACGACGACGACCTGGACAACCTCCTGGCCCAGAT
CGGCGACCAGTACGCCGACCTGTTCCTCGCCGCCAAGAACCTGTCCGACGCCATC
CTCCTGTCGGACATTCTCCGCGTCAACACCGAGATTACGAAGGCCCCTCTCTCCGC
CTCGATGATCAAGCGCTACGACGAGCACCACCAGGACCTGACCCTGCTCAAGGCC
CTGGTCCGCCAGCAGCTCCCCGAGAAGTACAAGGAGATCTTCTTTGACCAGAGCA
AGAACGGCTACGCCGGCTACATCGACGGCGGCGCTAGCCAAGAGGAGTTCTACAA
GTTTATCAAGCCCATTCTGGAGAAGATGGACGGCACGGAGGAGCTCCTGGTCAAG
CTCAACCGCGAGGACCTCCTGCGCAAGCAGCGCACCTTCGACAACGGCAGCATCC
CCCACCAGATTCACCTCGGCGAGCTGCACGCCATCCTCCGCCGACAAGAGGACTT
CTACCCCTTTCTCAAGGACAACCGCGAGAAGATCGAGAAGATTCTGACGTTCCGC
ATCCCCTACTACGTCGGCCCCCTGGCCCGCGGCAACAGCCGCTTTGCCTGGATGAC
CCGCAAGTCCGAGGAGACCATCACGCCCTGGAACTTCGAGGAAGTCGTCGACAA
GGGCGCCTCGGCCCAGTCCTTCATCGAGCGCATGACCAACTTTGACAAGAACCTG
CCCAACGAGAAGGTCCTCCCCAAGCACTCGCTCCTGTACGAGTACTTCACCGTCT
ACAACGAGCTCACGAAGGTCAAGTACGTCACCGAGGGCATGCGCAAGCCCGCCT
TCCTGTCGGGCGAGCAGAAGAAGGCCATCGTCGACCTCCTGTTTAAGACCAACCG
CAAGGTCACGGTCAAGCAGCTCAAGGAAGACTACTTCAAGAAGATTGAGTGCTTT
GACAGCGTCGAGATCTCCGGCGTCGAGGACCGCTTTAACGCCTCCCTGGGCACCT
ACCACGACCTCCTGAAGATCATTAAGGACAAGGACTTCCTGGACAACGAGGAGAA
CGAGGACATCCTCGAGGACATTGTCCTGACCCTCACGCTGTTTGAGGACCGCGAG
ATGATCGAGGAGCGCCTGAAGACGTACGCCCACCTCTTCGACGACAAGGTCATGA
AGCAGCTCAAGCGCCGCCGATACACCGGCTGGGGCCGCCTGAGCCGCAAGCTCAT
CAACGGCATTCGCGACAAGCAGTCGGGCAAGACGATCCTCGACTTCCTGAAGAGC
GACGGCTTCGCCAACCGCAACTTTATGCAGCTGATTCACGACGACTCCCTCACCTT
CAAGGAAGACATCCAGAAGGCCCAGGTCTCCGGCCAGGGCGACTCCCTGCACGA
GCACATCGCCAACCTCGCCGGCAGCCCCGCCATCAAGAAGGGCATTCTGCAGACC
GTCAAGGTCGTCGACGAGCTCGTCAAGGTCATGGGCCGCCACAAGCCCGAGAAC
ATCGTCATTGAGATGGCCCGCGAGAACCAGACCACGCAGAAGGGCCAGAAGAAC

AGCCGCGAGCGCATGAAGCGCATCGAGGAAGGCATCAAGGAGCTGGGCTCCCAG
ATCCTCAAGGAGCACCCCGTCGAGAACACCCAGCTGCAGAACGAGAAGCTCTAC
CTGTACTACCTCCAGAACGGCCGCGACATGTACGTCGACCAGGAGCTGGACATTA
ACCGCCTCTCGGACTACGACGTCGACCACATCGTCCCCCAGAGCTTCCTGAAGGA
CGACTCCATCGACAACAAGGTCCTCACCCGCAGCGACAAGAACCGCGGCAAGAG
CGACAACGTCCCCTCCGAGGAAGTCGTCAAGAAGATGAAGAACTACTGGCGCCA
GCTCCTGAACGCCAAGCTGATCACGCAGCGCAAGTTTGACAACCTCACCAAGGCC
GAGCGAGGCGGCCTCTCGGAGCTGGACAAGGCCGGCTTCATCAAGCGCCAGCTG
GTCGAGACCCGCCAGATCACGAAGCACGTCGCCCAGATTCTCGACTCGCGCATGA
ACACGAAGTACGACGAGAACGACAAGCTGATCCGCGAGGTCAAGGTCATTACCCT
GAAGTCGAAGCTCGTCAGCGACTTCCGCAAGGACTTCCAGTTTTACAAGGTCCGC
GAGATCAACAACTACCACCACGCCCACGACGCCTACCTCAACGCCGTCGTCGGCA
CCGCCCTGATCAAGAAGTACCCCAAGCTCGAGTCCGAGTTCGTCTACGGCGACTA
CAAGGTCTACGACGTCCGCAAGATGATCGCCAAGTCCGAGCAGGAGATTGGCAAG
GCCACCGCCAAGTACTTCTTTTACTCGAACATCATGAACTTCTTTAAGACCGAGAT
CACCCTCGCCAACGGCGAGATCCGCAAGCGCCCCCTCATTGAGACCAACGGCGAG
ACCGGCGAGATCGTCTGGGACAAGGGCCGCGACTTCGCCACCGTCCGCAAGGTCC
TCAGCATGCCCCAGGTCAACATCGTCAAGAAGACCGAGGTCCAGACGGGCGGCTT
CTCGAAGGAGAGCATTCTGCCCAAGCGCAACTCCGACAAGCTCATCGCCCGCAAG
AAGGACTGGGACCCCAAGAAGTACGGTGGCTTCGACTCCCCCACCGTCGCCTACT
CGGTCCTGGTCGTCGCCAAGGTCGAGAAGGGCAAGTCGAAGAAGCTCAAGAGCG
TCAAGGAGCTCCTGGGCATCACCATTATGGAGCGCAGCTCCTTCGAGAAGAACCC
CATCGACTTTCTCGAGGCCAAGGGCTACAAGGAAGTCAAGAAGGACCTGATCATT
AAGCTCCCCAAGTACTCCCTCTTCGAGCTGGAGAACGGCCGCAAGCGCATGCTCG
CCTCCGCCGGCGAGCTCCAGAAGGGCAACGAGCTCGCCCTGCCCAGCAAGTACG
TCAACTTCCTCTACCTGGCCAGCCACTACGAGAAGCTCAAGGGCTCCCCCGAGGA
CAACGAGCAGAAGCAGCTGTTTGTCGAGCAGCACAAGCACTACCTCGACGAGAT
CATTGAGCAGATTTCCGAGTTCTCGAAGCGCGTCATCCTGGCCGACGCCAACCTG
GACAAGGTCCTCAGCGCCTACAACAAGCACCGCGACAAGCCCATCCGCGAGCAG
GCCGAGAACATCATTCACCTCTTCACCCTGACCAACCTCGGCGCCCCCGCCGCCTT
CAAGTACTTTGACACCACGATCGACCGCAAGCGCTACACCTCGACGAAGGAAGTC
CTGGACGCCACCCTCATCCACCAGAGCATTACCGGCCTCTACGAGACGCGCATCG
ACCTCAGCCAGCTCGGCGGCGACTCCCGCGCCGA<u>CCCCAAGAAGAAGCGCAAGG</u>
<u>TC</u>TAA

The Flag-tag and SV40 nuclear localization sequence (NLS) sequences are indicated by lower case letters and underlines, respectively.

Sequence data S2: Nucleotide sequence of PtrPC-*neo*-TtrPC cassette

GC*TCTAGA*GCGCAATTAACCCTCACTAAAGGGAACAAAAGCTGGAGCTCCACCGC
GGTGGCGGCCGCGACGTAACTGATATTGAAGGAGCACTTTTTGGGCTTGGCTGGA
GCTAGTGGAGGTCAACAATGAATGCCTATTTTGGTTTAGTCGTCCAGGCGGTGAGC
ACAAAATTTGTGTCGTTTGACAAGATGGTTCATTTAGGCAACTGGTCAGATCAGCC
CCACTTGTAGCAGTAGCGGCGGCGCTCGAAGTGTGACTCTTATTAGCAGACAGGA
ACTAGGACATTATCATCATCTGCTGCTTGGTGCACGATAACTTGGTGCGTTTGTCAA

GCAAGGTAAGTGAACGACCCGGTCATACCTTCTTAAGTTCGCCCTTCCTCCCTTTG
TTTCAGATTCAATCTGACTTACCTATTCTACCCAAGCATCGAAGATATGATTGAACA
AGATGGATTGCACGCAGGTTCTCCGGCCGCTTGGGTGGAGAGGCTATTCGGCTATG
ACTGGGCACAACAGACAATCGGCTGCTCTGATGCCGCCGTGTTCCGGCTGTCAGC
GCAGGGGCGCCCGGTTCTTTTTGTCAAGACCGACCTGTCCGGTGCCCTGAATGAA
CTGCAGGACGAGGCAGCGCGGCTATCGTGGCTGGCCACGACGGGCGTTCCTTGCG
CAGCTGTGCTCGACGTTGTCACTGAAGCGGGAAGGGACTGGCTGCTATTGGGCGA
AGTGCCGGGGCAGGATCTCCTGTCATCTCACCTTGCTCCTGCCGAGAAAGTATCCA
TCATGGCTGATGCAATGCGGCGGCTGCATACGCTTGATCCGGCTACCTGCCCATTC
GACCACCAAGCGAAACATCGCATCGAGCGAGCACGTACTCGGATGGAAGCCGGTC
TTGTCGATCAGGATGATCTGGACGAAGAGCATCAGGGGCTCGCGCCAGCCGAACT
GTTCGCCAGGCTCAAGGCGCGCATGCCCGACGGCGAGGATCTCGTCGTGACCCAT
GGCGATGCCTGCTTGCCGAATATCATGGTGGAAAATGGCCGCTTTTCTGGATTCATC
GACTGTGGCCGGCTGGGTGTGGCGGACCGCTATCAGGACATAGCGTTGGCTACCC
GTGATATTGCTGAAGAGCTTGGCGGCGAATGGGCTGACCGCTTCCTCGTGCTTTAC
GGTATCGCCGCTCCCGATTCGCAGCGCATCGCCTTCTATCGCCTTCTTGACGAGTTC
TTCTGAATCAGTAGATGCCGACCGGGATCGATCCACTTAACGTTACTGAAATCATC
AAACAGCTTGACGAATCTGGATATAAGATCGTTGGTGTCGATGTCAGCTCCGGAGT
TGAGACAAATGGTGTTCAGGATCTCGATAAGATACGTTCATTTGTCCAAGCAGCAA
AGAGTGCCTTCTAGTGATTTAATAGCTCCATGTCAACAAGAATAAAACGCGTTTCG
GGTTTACCTCTTCCAGATACAGCTCAACTGCAATGCATTAATGCATTGGACCTCGCA
ACCCTAGTACGCCCTTCAGGCTCCGGCGAAGCAGAAGAATAGCTTAGCAGAGTCT
ATTTTCATTTTCGGGAGACGAGATCAAGCAGATCAACGGTCGTCAAGAGACCTAC
GAGACTGAGGAATCCGCTCTTGGCTCCACGCGACTATATATTTGTCTCTAATTGTAC
TTTGACATGCTCCTCTTCTTTACTCTGATAGCTTGACTATGAAAATTCCGTCACCAG
CCCTG*AAGCTT*GGG
The XbaI and HindIII sites are indicated by italic letters.

Sequence data S3: Nucleotide sequence of *U6* promoter of *A. oryzae* RIB40
TGGTTCACTTCTCTTTAGAAATCAACTGTGGGTTTTGCTTTTTGCTTCATTCTCTTTG
TCTTCTCCATCTTTGATCAAATCCTGGACTTTCTCAATCCCCAGCTAATTCAATCATA
GTCAGTTTTCTATTTTTATTATTTCTTTTTCTTTTGAAATGTGATTAACAACCAGTCC
GTTATATATCTTGTACCCAGATTACGCCCAACTCGTGCTCCTCAGCCACAAAGATAC
TCAATTGATAGCCAAGATACATACATACCACAAAGTAAGGACTCCATGCATTGAGTA
TTACTCATCGTATTCTAGACTACTCCAAAACTCAGCACATAGACAAACAATACGAA
CCTCGTCTAGGGGTGATTCAGAGGCGGCAAAGCGGGGTTTTCGCATTTGATGTTCC
TGGCACTTATGTAAGCCCACGCTTCCCGCTCAACTAAACCATCAGCCAATCAGACT
GCTCAGATTTATCTTTTGAAGGGTAAATAAATCATTGTAAAGAAGAACAAGT

Sequence data S4: Nucleotide sequence of *U6* promoter of *Nodulisporium* sp. (No. 65-12-7-1)
GTGTCCCATTAGACCCTTCTCCAGCAATGGCTGGTCTTGCTGCAGGCCGTTGTGGT
AGCAAACCACATATTAGGTAGTTAGGACCTGTCATAATCGTTGCAGTTCCACCAAT
GAAACTGACCCATCTAAGCACTTACGCCCGAGAGTAAAGAGCAATCGAGACACCA

AGGCGAAGCTGTCTGGCTTCTCTACTTACTGAATTGAGGCCGATGCATGCGAGCTT
ATCCTTGCGTCTCTTATCAGCGTATTGCCACGATGAATAGACTCTAAGTCAACCTGA
TGATTACAAAGTAGTCCCTAATGACAATGATGCATGAGTCGAGGTGCTGATAATGAT
GTTAGGTAGAAGTAGGAAGATTGATTAACATTGGCGATTCACACGTGGATCGATTG
TACTTTAAACAAACTGTGTGGGGCCCGGCCGGCTTGGATTACCCGGAATATTTCCG
ATTGGCTCCCCGCTATAGGGGTTATAAGACAAGATTGTCCTGTATAATTTC

Sequence data S5: Nucleotide sequence of *U6* terminator
TTTTTTTTTGAGCATTTATCAGCTTGATATAGAGGTAGGAATGTATGGAGGTGCAGA
ATGGCTATTTTGTTATTGGAGCGGGTTCGAAACGGAGGGCAGGAGACTTTTTCTAA
ATACGTCACGTGATATAGAGCTGCT

Sequence data S6: Nucleotide sequence of gRNA-*g3279* for *U6* promoter-mediated *in vivo* transcription
GAAGAGGGCGTGGAAACATAGTTTTAGAGCTAGAAATAGCAAGTTAAAATAAGGC
TAGTCCGTTATCAACTTGAAAAAGTGGCACCGAGTCGGTGC
Target sequence of *g3279* is underlined.

Sequence data S7: Nucleotide sequence of gRNA-*g3279* for *T7* promoter-mediated *in vitro* transcription
GGAGCCAATGCGCCAAGACCGTTTTAGAGCTAGAAATAGCAAGTTAAAATAAGGC
TAGTCCGTTATCAACTTGAAAAAGTGGCACCGAGTCGGTGC
Target sequence of *g3279* is underlined.

Sequence data S8: Nucleotide sequence of gRNA-*wA* for *T7* promoter-mediated *in vitro* transcription
GGATCTACTGGCGCGTCACCGTTTTAGAGCTAGAAATAGCAAGTTAAAATAAGGCT
AGTCCGTTATCAACTTGAAAAAGTGGCACCGAGTCGGTGC
Target sequence of *wA* is underlined.

Sequence data S9: Nucleotide sequence of gRNA-*HdaA* for *T7* promoter-mediated *in vitro* transcription
GGTCCCGTTCACCTCTGTTGGTTTTAGAGCTAGAAATAGCAAGTTAAAATAAGGCT
AGTCCGTTATCAACTTGAAAAAGTGGCACCGAGTCGGTGC
Target sequence of *HdaA* is underlined.

Table S1. Strains and plasmids used in this study

Strains/plasmids	Characteristic(s)	Source
**Strains**		
Escherichia coli		
DH5α	Host for general plasmid cloning	TaKaRa
**Fungi**		
Nodulisporium sp. (No. 65-12-7-1)	Wild-type strain, viridins producing	Lab stock
JN1001	Cas9-expressing *Nodulisporium* sp. (No. 65-12-7-1)	This study
JN1002	Hygromycin resistance clone generated by transformation with pBSKII-*toCas9-hph*	This study
JN1003	Hygromycin resistance clone generated by transformation with pBSKII-*toCas9-hph*	This study
JN1004	Hygromycin resistance clone generated by transformation with pBSKII-*toCas9-hph*	This study
JN1005	Hygromycin resistance clone generated by transformation with pBSKII-*toCas9-hph*	This study
JN1006	Hygromycin resistance clone generated by transformation with pBSKII-*toCas9-hph*	This study
JN1007	Hygromycin resistance clone generated by transformation with pBSKII-*toCas9-hph*	This study
A. oryzae NSAR1	Quadruple auxotrophic strain, (*niaD*$^-$, *sC*$^-$, Δ*argB*, *adeB*$^-$)	[1]
JA1001	Cas9-expressing *A. oryzae* NSAR1	This study
S. minima (No. 40-1-4-1)	Wild-type strain	Lab stock
JS1001	Cas9-expressing *S. minima* (No. 40-1-4-1)	This study
**Plasmids**		
pDHt/sk-Ppdc-*toCas9*-Tpdc	pDHt/sk containing *hph* cassette and	[2]

	Trichoderma reesei codon-optimized *cas9* (*toCas9*) gene cassette, (Kan^R)	
pDHt/sk-Ppdc-*toCas9-eGFP*-Tpdc	pDHt/sk containing *hph* cassette and *toCas9-eGFP* cassette, (Kan^R)	[2]
pBluescript SKII	*E. coli* cloning vector, (Amp^R)	Stratagene
pBSK**II**-PtrPC-EcoRV-TtrPC	pBluescript SKII containing the *A. nidulans trpC* promoter and terminator, (Amp^R)	This study
pBSKII-PtrPC-Flag-*toCas9*-TtrPC	pBSKII-PtrPC-EcoRV-TtrPC containing the Flag-tagged *toCas9* gene, (Amp^R)	This study
pBSKII-*toCas9-hph*	pBSKII-PtrPC-Flag-*toCas9*-TtrPC containing the *hph* cassette, (Amp^R)	This study
pUNAFNC9gwA1	pUNA containing *A. oryzae U6* promoter and terminator, gRNA scaffold and *A. oryzae* codon-optimized *cas9* gene (Amp^R)	[1]
pAdeA	Plasmid containing *adeB* maker gene cassette, (Amp^R)	Stratagene
pAdeA-*cas9*	pAdeA containing the *A. oryzae* codon-optimized *cas9* gene whose expression is regulated by *amyB* promoter, (Amp^R)	This study
pcDNA3.1	Mammalian expression plasmid containing *neo* maker gene cassette, (Amp^R)	Stratagene
pBSKII-PtrPC-*neo*-TtrPC	pBSKII-PtrPC-EcoRV-TtrPC containing *neo* gene, (Amp^R)	This study
pBSKII-PtrPC-*neo*-TtrPC-$U6_{Nod}$-gRNA-*g3279*	pBSKII-PtrPC-*neo*-TtrPC containing gRNA-*g3279* cassette whose expression is regulated by $U6_{Nod}$ promoter, (Amp^R)	This study

pBSKII-PtrPC-*neo*-TtrPC-*U6*$_{\mathrm{Asp}}$-gRNA-*g3279*	pBSKII-PtrPC-*neo*-TtrPC containing gRNA-*g3279* cassette whose expression is regulated by *U6*$_{\mathrm{Asp}}$ promoter, (*Amp*^{R})	This study
pUCm-T	*E. coli* cloning vector (*Amp*^{R})	Sangon Biotech Co., Ltd
pUCm-gRNAscaffold-*eGFP*	pUCm-T containing gRNA scaffold, (*Amp*^{R})	This study
pUCm-gRNA-*g3279*	pUCm-T containing gRNA-*g3279* cassette for *in vitro* preparation, (*Amp*^{R})	This study
pUCm-gRNA-*wA*	pUCm-T containing gRNA-*wA* cassette for *in vitro* preparation, (*Amp*^{R})	This study
pUCm-gRNA-*HdaA*	pUCm-T containing gRNA-*HdaA* cassette for *in vitro* preparation, (*Amp*^{R})	This study
pTAex3	Plasmid containing *argB* maker gene cassette	Stratagene

Table S2. Primers used in this study

Name	Sequence (5' to 3')	Experiment
PtrpC-XbaI-F	GC*TCTAGA*GCGCAATTAACCCTCACTAA	Cloning of *trPC* promoter
PtrpC-R	TTCGATGCTTGGGTAGAATAG	Cloning of *trPC* promoter
TtrpC-EcoRV-F	CTATTCTACCCAAGCATCGAA*GATATC*AGTAGATGCCGACCGGGATCG	Cloning of *trPC* terminator
TtrpC-HindШ-R	CCC*AAGCTT*CAGGGCTGGTGACGGAATTTTCATAG	Cloning of *trPC* terminator
neo-F	ATGATTGAACAAGATGGATTG	Cloning of *neo* gene
neo-R	TCAGAAGAACTCGTCAAGAAG	Cloning of *neo* gene

PtrpC-AanI-F	CCGAA*TTATAA*GCGCAATTAACCCTCACTAA	Cloning of PtrpC-*hph*-TtrpC
TtrpC-AanI-R	CCGAA*TTATAA*CAGGGCTGGTGACGGAATTTTC	Cloning of PtrpC-*hph*-TtrpC
Flag-*cas9*-F	ATGGATTACAAGGATGACGACGATAAGCCCAAGAAGAAGCGCAAGGTCGACAAGAAGTACAGCATTG	Cloning of *T. reesei* codon-optimized *cas9* gene
cas9-R	TTAGACCTTGCGCTTCTTCTTGGG	Cloning of *T. reesei* codon-optimized *cas9* gene
amy-F	GCAGGTCGACTCTAGATGGTGTTTTGATC	Cloning of *A. oryzae* codon-optimized *cas9* gene
amy-R	TAGTAGATCCTCTAGGGATCCTTTCCTATAATAG	Cloning of *A. oryzae* codon-optimized *cas9* gene
*U6*p-F1	GTGTCCCATTAGACCCTTC	Cloning of *U6* promoter from *Nodulisporium* sp. (No. 65-12-7-1)
*U6*P-R1	GCGTGTCATCCTTAGTGCAG	Cloning of *U6* promoter from *Nodulisporium* sp. (No. 65-12-7-1)
*U6*p-AanI-F2	CCGAA*TTATAA*TAATGCCGGCTCATTCAAAC	Cloning of *U6* promoter from *A. oryzae* RIB40
*U6*p-R2	ACTTGTTCTTCTTTACAATG	Cloning of *U6* promoter from *A. oryzae* RIB40
gRNA-*U6*t-F	CATTGTAAAGAAGAACAAGTGAAGAGGGCGTGGAAACATAGTTTTAGAGCTAGAA	Cloning of gRNA scaffold and *U6*

	ATAGC	terminator
*U6*t-AanI-R	CCGAA*TTATAA*AGCAGCTCTATATCACGTGAC	Cloning of gRNA scaffold and *U6* terminator
gRNA-*g3279*-F	TAATACGACTCACTATAGGAGCCAATGCGCCAAGACCGTTTTAGAGCTAGAAATAGC	Construction of pUCm-gRNA-*g3279*
gRNA-*wA*-F	TAATACGACTCACTATAGGATCTACTGGCGCGTCACCGTTTTAGAGCTAGAAATAGC	Construction of pUCm-gRNA-*wA*
gRNA-*HdaA*-F	TAATACGACTCACTATAGGTCCCGTTCACCTCTGTTGGTTTTAGAGCTAGAAATAGC	Construction of pUCm-gRNA-*HdaA*
eGFP-R	TTACACCTTCCTCTTCTTC	Construction of pUCm-gRNA-scaffold-*eGFP*
g3279-F	TTGCGAACCGACGACGAACC	Amplification of the DNA regions surrounding the target site of *g3279*
g3279-R	TCCGGCTCTGGGAGACGAAG	Amplification of the DNA regions surrounding the target site of *g3279*
wA-F	TTCCAGAGATGCTTTCACGC	Amplification of the DNA regions surrounding the target site of *wA*
wA-R	TCCGTAGTAGCTATCTCAGG	Amplification of the DNA regions

		surrounding the target site of *wA*
HdaA-F	ATGGATCAAGAAGATTTCGAC	Amplification of the DNA regions surrounding the target site of *HdaA*
HdaA-R	TCAGGATGGATGTCATTAGC	Amplification of the DNA regions surrounding the target site of *HdaA*
pUCm-F	TCGCGCGTTTCGGTGATGAC	Amplification of transcription templates
gRNA-R	AAAAGCACCGACTCGGTGCC	Amplification of transcription templates
GAPDH-F	AATGGCAAGCTCACCGGAATG	RT-PCR analysis of GAPDH
GAPDH-R	GTTGGTGTTGCCGTTCAAGTC	RT-PCR analysis of GAPDH
cas9-F1	GGAGATTTTCTCCAACGAGA	Amplification of *cas9* gene
cas9-R1	TAATGGGGTTCTCCTCAAAG	Amplification of *cas9* gene
argB-F	AAGCTTTATTTCGCGGTTTTTTG	Amplification of *argB* maker gene cassette
argB-R	GTCGACCTACAGCCATTGCG	Amplification of *argB* maker gene cassette

The restriction sites and the SV40 NLS sequence are indicated by italic letters and underlins, respectively.

Table S3. Nucleotide sequences of the random DNA fragment attached to the *neo* cassette in *g3279* mutants in Fig. 3e

Clones	Random fragment sequence (left)	Random fragment sequence (right)
1	T	
2	GCTCTAGAGCGCAATTAACCCTC<br>ACTAAAGGGAACAAAAGCTGGA<br>GCTCCACCGCGGTGGCGGCC	
4		CCGTAACGAGCACATTCCCGATTTG<br>GATCAGCGTGCCTAGCAAGGAGAT<br>CCACCGCTGGA
7	GCTCTAGAGCGCAATTAACCCTC<br>ACTAAAGGGAACAAAAGCTGGA<br>GCTCCACCGCGGTGGCGGCC	
9	CTAGAGCGCAATTAACCCTCACT<br>AAAGGGAACAAAAGCTGGAGCT<br>CCACCGCGGTGGCGGCCGCTCTA<br>GAGCGCAATTAACCCTCACTAAA<br>GGGAACAAAAGCTGGAGCTCCA<br>CCGCGGTGGCGGCC	
11		GGCCGCCACCGCGGTGGAGCTCCA<br>GCTTTTGTTCCCTTTAGT
12	TGGATTGCACGCAGGTTCTCCGG<br>CCGCTTGGG	GGCCGCCACG

Fig. S1. Analysis of the integration of *hph* gene into the genome of the clones transformed with pBSKII-*tocas9-hph*.

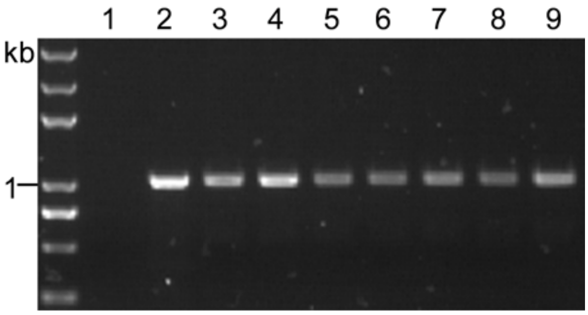

Lane 1: wild-type; lane 2: pBSKII-*tocas9-hph*; lane 3: JN1002; lane 4: JN1001; lane 5: JN1003; lane 6: JN1004; lane 7: JN1005; lane 8: JN1006; lane 9: JN1007.

Fig. S2. HPLC analysis of the secondary metabolites of wild-type and JN1001.

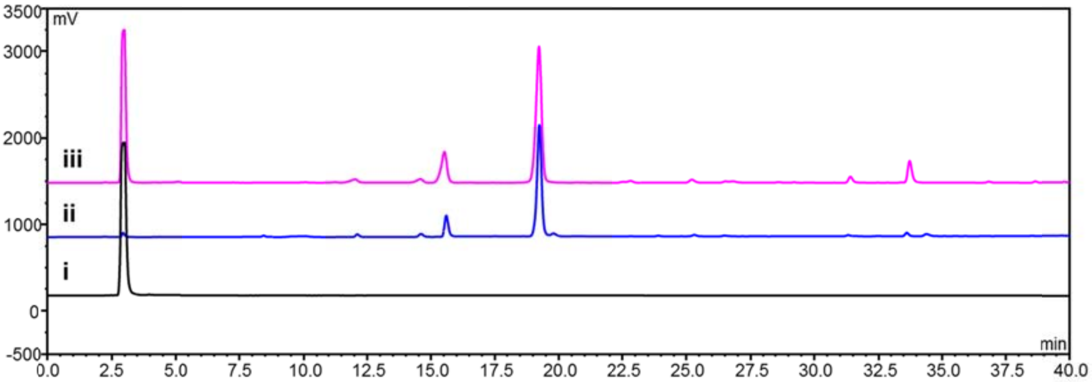

(i) Culture medium; (ii) wild-type; (iii) JN1001. Fermentation was performed on maltose medium with shaking at 200 rpm at 18 °C for 2 d.

Fig. S3. Sequence alignment of *U6* snRNA genes.

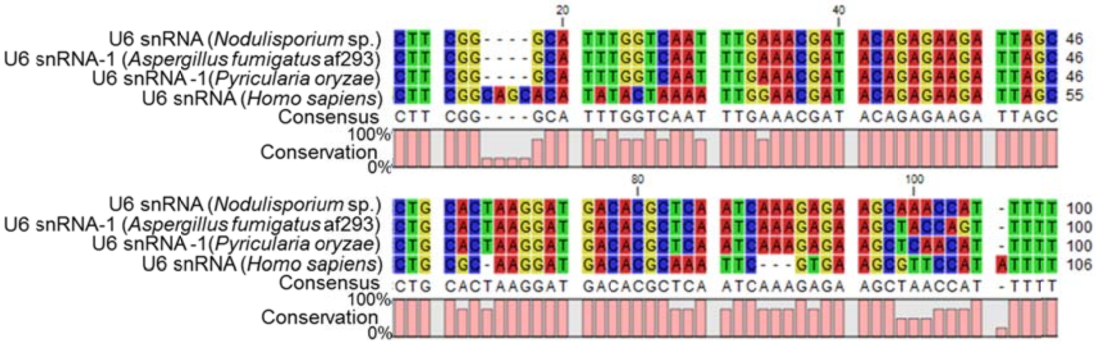

Fig. S4. CRISPR-Cas9-based gene disruption by *U6* promoter driving gRNA expression.

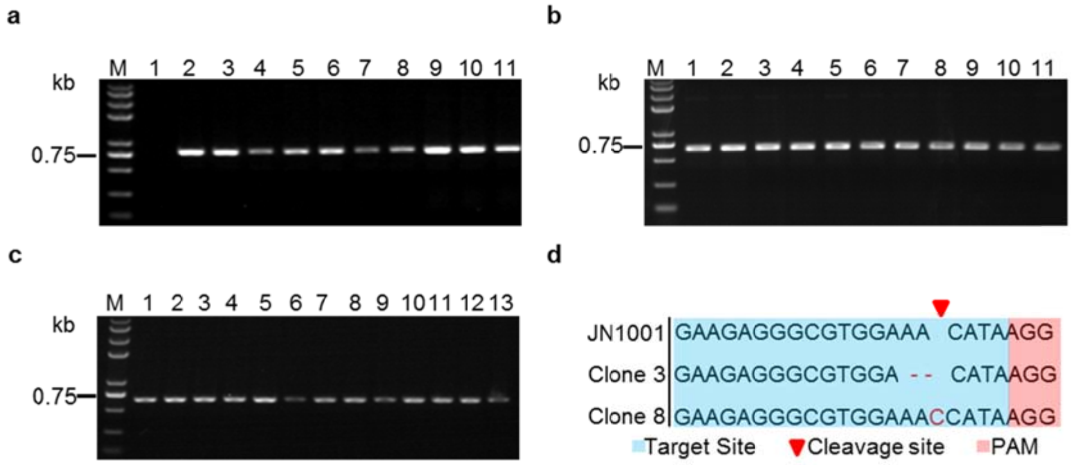

(a) Analysis of the integration of *neo* gene into the genome of ten G418-resistance clones generated by $U6_{\mathrm{Nod}}$ promoter driving CRISPR-Cas9 system. (b) PCR amplification of the DNA regions containing the target site of the ten clones described in (a) using primers flanking the target site. (M: DNA marker; lane 1: JN1001; lane 2-11: G418-resistance clones (No. 1-10)). (c) PCR amplification of the DNA regions containing the target site of the twelve clones generated by $U6_{\mathrm{Asp}}$ promoter driving CRISPR-Cas9 system. (M: DNA marker; lane 1: JN1001; lane 2-13: G418-resistance clones (No. 1-12)). (d) Sequence analysis of the PCR products described in (c) revealed that two clones had mutations at the target site.

Fig. S5. Both Cas9 and *in vitro* gRNA are essential for mutagenesis

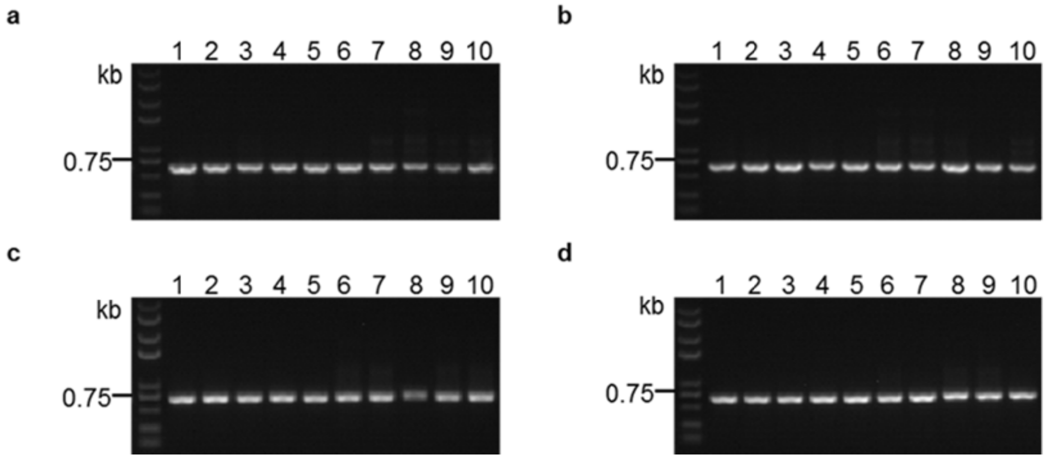

(a) PCR amplification of the DNA regions containing the target site of the ten clones generated by transformation of wild-type with linear neo cassette; (b) PCR amplification of the DNA regions containing the target site of the ten clones generated by transformation of

wild-type with circular plasmid; (c) PCR amplification of the DNA regions containing the target site of the ten clones generated by transformation of JN1001 with linear neo cassette; (d) PCR amplification of the DNA regions containing the target site of the ten clones generated by transformation of JN1001 with circular plasmid. ((a)-(d), lane 1-10: G418-resistance clones (No. 1-10))

Fig. S6. Analysis of the effects of usage amount of linear *neo* cassette on the mutation efficiency.

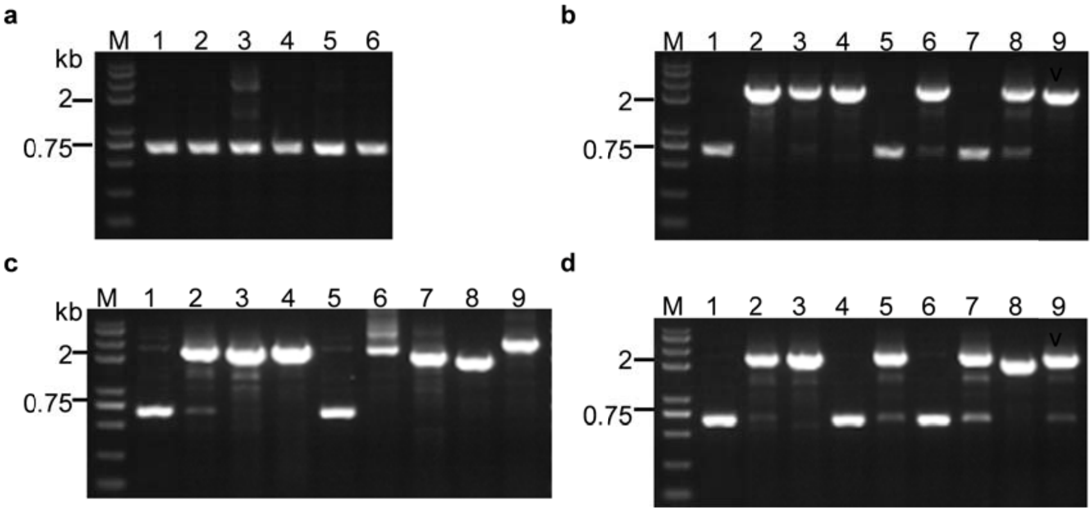

PCR amplification of the DNA regions surrounding the target site of *g3279* in the transformants generated using different amount of linear *neo* cassette. (a), 0.5 μg; (b), 2 μg; (c), 3 μg; (d), 4 μg. (M: DNA Marker; lane 1: JN1001; lane 2-9: G418-resistance clones (No. 1-8))

References

1. Katayama, T. et al. Development of a genome editing technique using the CRISPR/Cas9 system in the industrial filamentous fungus *Aspergillus oryzae*. *Biotechnol Lett* **38**, 637-642 (2015).

2. Liu, R., Chen, L., Jiang, Y.P. , Zhou, Z.H & Zou, G. Efficient genome editing in filamentous fungus *Trichoderma reesei* using the CRISPR/Cas9 system. *Cell Discov* **1**, 15007 (2015).
